# Unraveling the link between metabolic dysfunction-associated steatotic liver disease and osteoporosis: a bridging function of gut microbiota

**DOI:** 10.3389/fendo.2025.1543003

**Published:** 2025-04-01

**Authors:** Jing Zhang, Zhen Sun, Lili Xu, Yunyang Wang, Yangang Wang, Bingzi Dong

**Affiliations:** ^1^ Department of Endocrinology and Metabolism, The Affiliated Hospital of Qingdao University, Qingdao, China; ^2^ Department of Neurology, The Affiliated Hospital of Qingdao University, Qingdao, China

**Keywords:** metabolic dysfunction-associated steatotic liver disease (MASLD), osteoporosis, gut microbiota, metabolism, bone-gut axis

## Abstract

This review examines the strong association between metabolic dysfunction-associated steatotic liver disease (MASLD) and osteoporosis (OP), with a particular focus on the role of gut microbiota in linking these two disorders. Both MASLD and OP are closely linked to metabolic syndrome, and their pathogenesis involves multiple factors, such as inflammatory response, insulin resistance, altered intestinal permeability, and estrogen deficiency. Dysregulation of gut microbiota not only affects hepatic fat accumulation and bone metabolism disorders through metabolites, such as short-chain fatty acids, but also exacerbates systemic chronic inflammation by impairing the intestinal barrier function, thus accelerating the progression of both diseases. This article summarizes recent studies that highlight the central role of gut microbiota as a co-morbid factor in MASLD and OP, offering new perspectives for future diagnostic and therapeutic strategies.

## 1 Introduction

The gut microbiota (GM), comprising more than 10^^^13 to 10^^^14 microorganisms in the human gastrointestinal tract, contains approximately 150 times the number of genes found in the human genome (1). This richest and most complex microbial ecosystem in the human body interacts with the host and plays a key role in regulating metabolism and the immune system, making it one of the central factors affecting human health. Imbalances in GM have been linked to a number of diseases including diabetes, obesity, Alzheimer’s disease, rheumatoid arthritis, and multiple sclerosis (2). Metabolic dysfunction-associated steatotic liver disease (MASLD) has garnered significant attention in recent years and is defined by the presence of hepatic steatosis along with any of the three metabolic disorders: overweight/obesity, diabetes mellitus, and metabolic dysfunction (3). Compared to non-alcoholic fatty liver disease (NAFLD), the definition of MASLD places more emphasis on the role of metabolic dysfunction in fatty liver disease rather than just excluding alcoholic liver disease. In 2023, three major multinational liver associations proposed that MASLD should replace the term NAFLD (4). The etiology of MASLD is complex and closely related to insulin resistance and metabolic dysfunction. With the global rise in obesity, type 2 diabetes mellitus, and metabolic syndrome, the burden of MASLD has significantly increased. Its global prevalence rose from 25.3% (1990–2006) to 38.2% (2016-2019), marking a nearly 50% increase over the past three decades (5), making it now the most common cause of chronic liver disease (6). Osteoporosis (OP) is another chronic metabolic bone disease that is gaining prominence with the aging of the population and is characterized by a combination of factors that lead to a decrease in bone mineral density and loss of bone tissue microarchitecture, resulting in increased bone fragility and fracture susceptibility (7). Its pathogenesis may be related to factors such as genetics, changes in hormone levels, nutritional deficiencies, drug effects, and unhealthy lifestyles (8). According to the World Health Organization(WHO), more than 200 million people worldwide are affected by OP (9), and the disease has become one of the major global public health problems.

A growing number of studies have identified multiple co-morbidities between MASLD and OP, with GM playing an important role. GM regulates immune responses and inflammation, and can also affect energy balance, insulin sensitivity, and lipid metabolism, with complex bidirectional crosstalk with multiple systems throughout the body. Abnormal changes in GM may lead to liver fat accumulation and bone metabolism disorders. This paper discusses the pathogenic mechanisms of GM as a co-morbid factor in MASLD and OP from multiple perspectives based on the concepts of the gut-liver axis and the gut-bone axis, and summarizes the bridging role that GM and its metabolite alterations play between the two diseases, with a view to providing references to the treatment of the disease and future research. Specific mechanisms include inflammatory response, estrogen deficiency, short-chain fatty acids (SCFAs), and compromised intestinal epithelial permeability.

## 2 Underlying mechanisms

### 2.1 Inflammatory response

**Figure 1:**
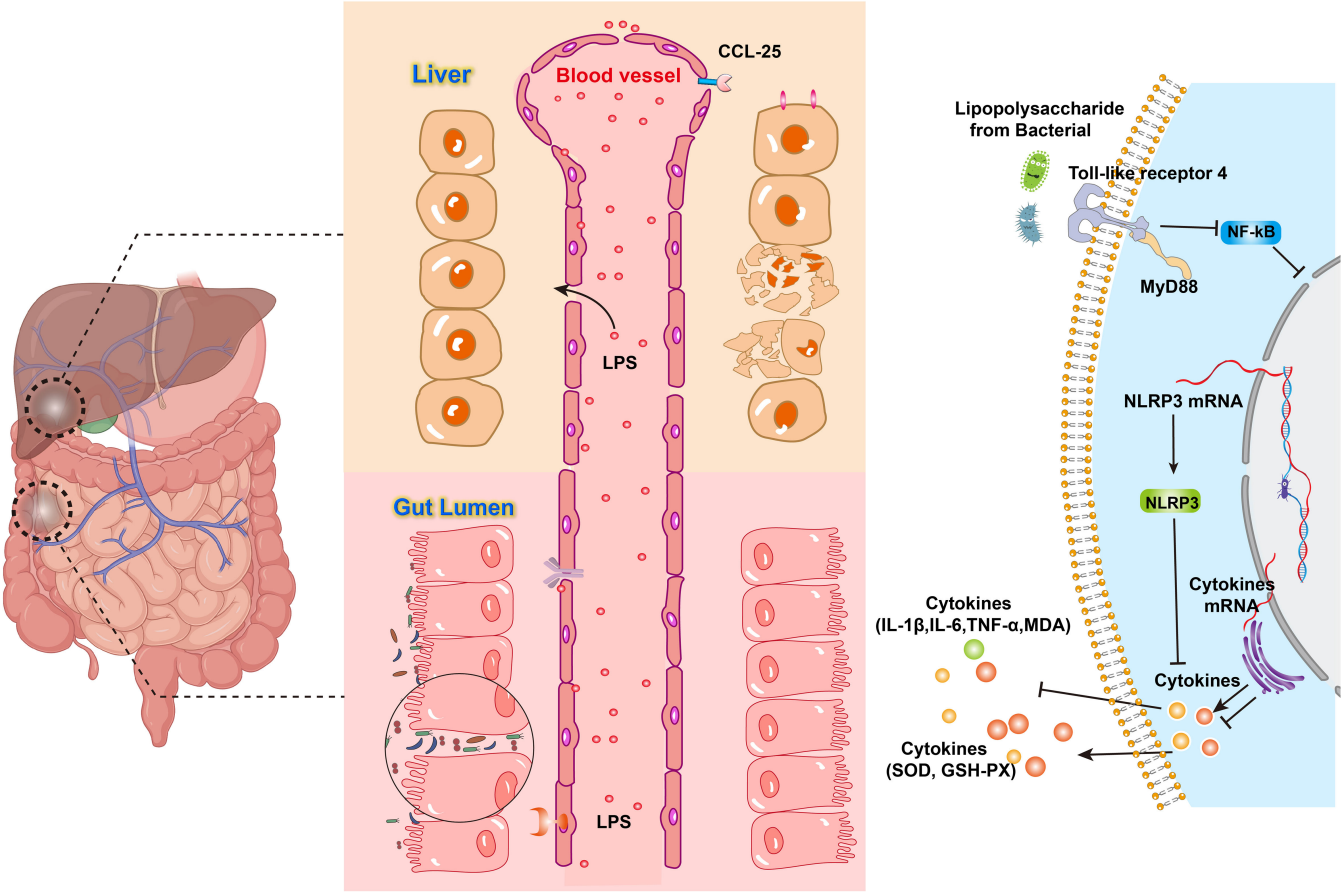
LPS acts on TLR to activate the NF-κB pathway and produce cytokines.

Pattern recognition receptors NOD1 and NOD2 in the cytoplasm of intestinal epithelial cells recognize peptidoglycan, a component of the bacterial cell wall, which in turn activates the innate immune system, increases the number of γδ T cells, and promotes the intestinal release of the pro-inflammatory cytokine IL-17 (10). GM imbalance also regulates the body’s inflammatory response through degradation of mucins and inhibition of mucin production, and the reduction of mucins activates the NF-κB pathway in cells and increases the production of pro-inflammatory cytokines (e.g., TNF-α, IL-1β, and interferon γ) (11). LPS produced by Gram-negative intestinal microbiota is a potent systemic activator of the inflammatory response and enters the body’s circulation in a state known as “metabolic endotoxemia” (12). Toll-like receptors (TLR) are pattern recognition receptors that activate the innate immune system. LPS initiates TLR4 signaling and phosphorylates IL-1 receptor-associated kinase (IRAK). Subsequently, IKKβ protein is activated, which phosphorylates IκB protein, leading to IκB ubiquitination and degradation. As a result, NF-κB proteins are released and translocated to the nucleus. These NF-κB proteins ultimately induce gene expression of inflammatory cytokines, including TNF-α, IL-1β, and IL-6 (**Figure 1**) (13), leading to a systemic chronic inflammatory state. Hepatocytes, Kupffer cells, and hepatic stellate cells, all express TLRs (14), with TLR4 being one of the major TLRs involved in the development of NAFLD (15). Inflammatory factors such as TNF-α and IL-1 can promote osteoclast activity by enhancing RANKL expression (16), inhibiting bone formation and accelerating bone resorption.

### 2.2 Estrogen deficiency

The relationship between estrogen and NAFLD and OP is one of the current hot topics in medical research. GM secretes β-glucuronidase, an enzyme that catabolizes estrogen into its active form. When this process is impaired by GM dysregulation, the reduction in catabolism leads to a decrease in circulating estrogen (17). Many studies have shown that estrogen deficiency leads to disorders of hepatic lipid metabolism and promotes the onset and progression of NAFLD. In animal models lacking endogenous estrogen, such as aromatase-deficient mice and ovariectomized mice (OVX), researchers observed significant hepatic fat accumulation, steatosis, and associated abnormal gene expression, which were effectively reversed by estradiol supplementation (18). This suggests that estrogen receptor-mediated signaling pathways play an important role in hepatic lipid metabolism. Further human studies also suggest that estrogen deficiency may exacerbate the progression of NAFLD. In a study of 875 women, the cumulative risk of NAFLD at 1, 2, and 5 years after oophorectomy was 14.1%, 20.5%, and 38.4%, respectively, and the cumulative risk was higher at younger ages (19). Estrogen stimulates osteoprotegerin (OPG) production, inhibits IL-1 and TNF-α release, promotes osteoclast apoptosis, and inhibits osteoclastogenesis. It also activates the Wnt signaling pathway to increase osteogenesis. In contrast, estrogen deficiency leads to bone resorption and decoupling of bone formation, which leads to bone loss (20). A study by Guan et al., 2023 found that estrogen deficiency in OVX mice leads to increased LPS levels and triggers GM dysregulation, especially increasing the proportion of bacteroides. This was ameliorated by antibiotic treatment, which increased bone density and improved bone structure in OVX mice (21). In another study, researchers found increased intestinal mucosal permeability and cytokines (TNFα and IL-17) in the small intestine of OVX mice, but the effects of estrogen deficiency on intestinal permeability and cytokine dysregulation were eliminated in germ-free mice (22), suggesting that estrogen deficiency-induced bone loss may be mediated through GM.

### 2.3 SCFAs

SCFAs are one of the important components of intestinal metabolites, mainly composed of carboxylic acids and small hydrocarbon chains, which can be produced by intestinal commensal bacteria through the fermentation of indigestible carbohydrates in food (23), and have been recognized as a key bridge connecting the gut microbiome to human health. It has been shown that SCFAs exert multiple beneficial regulatory effects on liver and bone by acting on G protein-coupled receptors (GPCRs) on different cells. Firstly, SCFAs act on terminal L cells in the small intestine, prompting them to release GLP-1, enhancing glucose-dependent insulin secretion and improving insulin resistance, which reduces appetite and delays gastric emptying, and contributes to the reduction of body weight and body fat, which are the main factors in the formation of MASLD. It has been suggested that GLP-1 may also act directly in the liver to reduce liver inflammation and slow down the fibrosis process (24). Secondly, SCFAs can inhibit lipolysis, reduce the level of free fatty acids in the blood, and promote adipocyte differentiation after acting on GPR43 and GPR41 in adipocytes (25). Some animal experiments found that acetate positively regulated adipocyte differentiation and lipid deposition by activating the MAPKs and mTOR signaling pathways (up-regulation of the expression of genes such as PPAR-γ, C/EBP-α, and SREBP-1) and inhibiting the AMPK signaling pathway (down-regulation of the expression of genes such as HSL, CPT-1, and AMPK-α) (26).

Upon binding to GPR41, GPR43 and GPR109a expressed on osteoblasts and osteoclasts, SCFAs activate multiple intracellular signaling pathways, such as the ERK/MAPK, JNK, and p38 MAP kinase pathways, thereby promoting osteoblast differentiation and function, while inhibiting osteoclast formation and activity, and this dual action is the basis for the potential of SCFAs to prevent bone loss and enhance bone density. Specifically, propionic acid and butyric acid inhibit histone deacetylase (HDAC) (27, 28), which increases histone acetylation levels, alters chromatin structure, and promotes the expression of genes associated with osteogenesis. For example, butyric acid increases the transcriptional activity of the osteogenesis-related factor Runx2 (29), which promotes osteoblast differentiation and mineralization activity. This epigenetic regulatory mechanism provides an important molecular basis for the role of SCFAs in bone health and disease. In addition, Kim et al. showed that SCFAs increase the solubility of minerals in the gut by lowering the intestinal pH, thus making calcium more readily absorbed by the body (30). Butyric acid enhances calcium absorption by increasing the surface area of the intestine available for absorption (31). However, when GM is dysregulated, the balance between the production and metabolism of SCFAs is disrupted, leading to disturbances in their concentration and distribution in the body and exacerbating the risk of NAFLD and OP.

### 2.4 Compromised Intestinal Epithelial Permeability

The intestinal epithelium acts as a barrier to prevent intestinal microorganisms and their metabolites from entering the circulation (32), and when the intestinal mucosal barrier is compromised, “leaky gut” can lead to bacterial translocation and toxin invasion. Using a junctional adhesion molecule A (JAM-A) knockout mouse model, which has a defective intestinal epithelial barrier, Rahman et al. demonstrated a link between increased intestinal permeability and hepatic inflammation. Mice with defective intestinal epithelial permeability developed more severe steatohepatitis compared to controls. This study also found that this inflammation was effectively alleviated by administering antibiotics, further emphasizing the central role of microbial translocation in triggering the inflammatory response in the liver (33). The concept of the “bone-gut axis” is an innovative and promising framework to explain the bidirectional communication between the GM and bone metabolism. GM is involved in the regulation of bone mineral density through the production of SCFAs, increasing intestinal permeability, and influencing calcium and vitamin D absorption (34). In turn, bone-derived factors such as osteocalcin may also influence gut health. Harris et al. found that chemically increasing gut barrier permeability in young mice resulted in decreased bone density and growth retardation compared to control mice (35). Zhang et al. treated ovx mice with faecal microbiota transplantation(FMT), which increased the expression of tight junction proteins (ZO-1 and Occludin), which guard the intestinal mucosal barrier, and reduced intestinal permeability, thereby decreasing the release of pro-osteoclastogenic cytokines (IL-1β and TNF-α) release (30).

## 3 Discussion

In the exploration of the relationship between MASLD and OP, the metabolic syndrome provides an important framework that reveals the complex interplay between metabolic factors and the two diseases. Metabolic syndrome is a group of clinical syndromes with aggregated onset of obesity, hyperglycemia, dyslipidemia, and hypertension that severely affect the health of the body. It has been suggested that MASLD is the hepatic manifestation of the metabolic syndrome (36), and that factors such as insulin resistance, dyslipidemia, and inflammatory states play a central role in the development of MASLD. Again, these factors are critical to the occurrence of OP. Insulin resistance may accelerate bone loss by affecting insulin signaling pathways in bone metabolism (37), whereas inflammation and oxidative stress directly affect bone resorption and formation, leading to reduced bone density. In addition to these co-morbid clinical features, patients with both diseases often share unhealthy lifestyle factors, such as poor dietary habits and lack of exercise. There have been many studies confirming the relationship between MASLD and OP: a population-based retrospective cohort study has confirmed that MASLD may increase the risk of developing OP (38); Wang et al. demonstrated that bone loss was significantly increased in mice with fatty liver induced by a high-fat diet and was strongly associated with molecules such as TNF-α, IL-6, and IGF-1 (39). In the previous section, we discussed in detail the effects of GM imbalance on MASLD and OP, which act as important co-morbid factors involved in the development of both diseases by influencing the metabolic processes, immune responses, etc., of the host.

Among the effects of GM imbalance, insulin resistance (40) is the most central mechanism linking the two diseases. It is not only a central feature of type 2 diabetes, but also closely associated with the association between MASLD and OP. Insulin resistance leads to abnormal lipid metabolism and increased circulating free fatty acids, which promotes hepatic fat accumulation and lipotoxic lipid formation, which in turn causes cellular oxidative stress, which further promotes the activation of inflammatory vesicles and the production of inflammatory factors, and ultimately leads to a series of consequences such as systemic chronic low-grade inflammatory states and disorders of glucose and lipid metabolism (41). Chronic low-grade inflammatory state indirectly promotes insulin resistance through activation of inflammatory pathways (e.g., NF-κB pathway). These pathways are activated in inflammatory cells in liver, muscle, and adipose tissue, leading to a vicious cycle of diminished response to insulin in these tissues, exacerbating insulin resistance. In a state of insulin resistance, insulin signaling pathways are impaired, including reduced phosphorylation of insulin receptors and their downstream signaling molecules, such as Akt proteins (42). This signaling impairment causes osteoblasts to fail to respond properly to insulin, thus affecting bone production. Some studies have shown that insulin resistance increases the risk of OP and fractures by affecting the bone turnover process (43); A randomized controlled trial suggests that insulin resistance may inhibit muscle-dependent bone gain by impairing the IGF-1 signaling pathway (44). In conclusion, insulin resistance is not only an important precursor state of type 2 diabetes, but also closely associated with the development of MASLD and OP. It has been shown that GLP-1 receptor agonist (GLP-1RA) significantly alter the composition of GM by increasing the abundance of beneficial bacteria (e.g., Bifidobacterium and Lactobacillus) while decreasing the proportion of pro-inflammatory flora (e.g., Enterobacteriaceae), which promotes the production of SCFAs and enhances GM function (45). This modulatory effect of GM not only directly improves metabolic and inflammatory status, but may also play a key role in MAFLD and OP by modulating downstream signalling pathways. GLP-1RAs improves a key step in the insulin signaling pathway - enhancement of PI3K/Akt pathway activity (46), thereby enhancing the cellular response to insulin, and they have been shown to be effective for fatty liver (47). GLP-1RAs acts on GLP-1 receptors on osteoblasts to activate PI3K/AKT and cAMP/PKA signaling, thereby promoting osteogenic differentiation and bone formation (48). In addition, the role of the GLP-1R/PI3K/AKT signaling pathway was also demonstrated in bone marrow mesenchymal stem cell experiments: activation of GLP-1 receptors by exenatide promotes osteogenic differentiation, increases bone formation and the number of osteoblasts, and inhibits lipidogenic differentiation of bone marrow mesenchymal stem cells (49).

Inflammation also bridges the gap between the two. Alterations in GM composition and metabolites can affect the barrier function of the intestinal mucosa. Dysregulation of GM causes damage to the intestinal barrier and a decrease in its protection against pathogens and endogenous toxins, leading to easier entry of LPS and other pro-inflammatory bacterial products into the bloodstream, which activates the immune system and prompts the production of inflammatory cells that produce a variety of inflammatory factors, such as TNF-α, IL-1β and IL -6. In the development of MASLD, LPS and other metabolites enter the bloodstream through a compromised intestinal barrier and enter the liver via the portal vein, activating immune cells in the liver and promoting inflammatory responses and liver fibrosis. Similarly, inflammation and immune responses play a crucial role in the regulation of osteoclastogenesis and bone remodeling processes. GM, by stimulating an inflammatory response, ultimately acts to inhibit bone formation and accelerate bone resorption. Several studies have confirmed the strong link between inflammation and OP. The NF-κB signaling pathway mediates increased expression of IL-20, which promotes osteoclast differentiation and activity and increases bone resorption (50); IL-7 shows the ability to inhibit basal and BMP2-induced bone formation in cranial organ cultures *in vitro* and in *in vivo* experiments in mice (51); IL-7 directly induces osteoclast formation through STAT5 activation, independent of the RANKL pathway (52).

Recent studies have identified specific changes in the composition of the GM associated with the progression of MASLD and OP. Significant reductions in the abundance of beneficial bacteria such as mucinophilic Akkermansia muciniphila have been observed in patients with MASLD and OP. This is a mucin-degrading bacterium that plays a key role in maintaining gut barrier function and reducing systemic inflammation. Depletion of Akkermansia muciniphila is associated with increased intestinal permeability allowing translocation of bacterial products such as LPS into the blood stream, which exacerbates hepatic inflammation and bone loss (53, 54). In contrast, an increase in pro-inflammatory bacteria, such as Escherichia coli and Enterobacteriaceae, is associated with disease progression (55).

While the GM has been extensively studied, emerging evidence highlights the significant roles of the mycobiome and virome in the pathogenesis of MASLD and OP. Dysbiosis of the mycobiome, particularly Candida overgrowth, has been linked to increased intestinal permeability, systemic inflammation, and metabolic disorders such as insulin resistance and obesity, all of which contribute to MASLD and OP (55, 56). Mycobiome components like β-glucans can activate immune cells and promote pro-inflammatory cytokines (TNF-α, IL-6), exacerbating liver inflammation and bone resorption. Similarly, the gut virome plays a crucial role in regulating bacterial populations and metabolic pathways. Bacteriophages influence the production of SCFAs, while eukaryotic viruses like norovirus can affect immune responses and contribute to metabolic dysfunction (57). Interactions between the mycobiome, virome, and GM underscore the complexity of the gut ecosystem and suggest potential therapeutic approaches, such as antifungal agents and phage therapy, to address both MASLD and OP.

It has been found that anti-osteoporotic drugs (e.g., bisphosphonates) not only directly inhibit osteoclast activity, but also indirectly affect bone metabolism by modulating GM. For example, an animal experiments have shown that bisphosphonate treatment can increase the abundance of beneficial bacteria, such as lactobacilli and bifidobacteria, in the intestine, reduce the number of pro-inflammatory bacteria and the release of pro-inflammatory cytokines (e.g., TNF-α and IL-6), and indirectly inhibit bone resorption; increase the production of SCFAs, activate the GPR41 and GPR43 receptors, promote osteoclast differentiation and inhibit osteoclastogenesis, which, in turn, improves bone density (58). This provides a new perspective for the treatment of OP.

Understanding the role of GM in MASLD and OP provides new perspectives for the development of shared preventive and therapeutic strategies. Existing therapies such as probiotics, prebiotics and FMT may exert their beneficial effects in part by modulating the gut microbiota. Ferrere et al. demonstrated that pectin prevents liver injury in a rodent model by restoring levels of Mycobacterium anthropomorphis (59). Britton et al. showed that treatment with the probiotic Lactobacillus Royale altered the microbial community in the gut of OVX mice, resulting in a decrease in osteoclastogenesis and an increase in bone marrow CD4+ T-lymphocytes, significantly preventing their bone loss (60). FMT is a promising therapeutic approach for MASLD and OP. In MASLD, FMT from healthy donors reduces hepatic inflammation and fibrosis by restoring GM homeostasis and decreasing levels of the pro-inflammatory bacterial product LPS (59). In animal models of OP, FMT increases the abundance of SCFAs-producing genera and decreases intestinal permeability, which reduces the release of pro-osteoclastogenic cytokines and ultimately improves bone density (30). These findings provide new clues for future treatments, and targeting GM may lead to new breakthroughs for both diseases.

Despite the potential of probiotics and dietary interventions to modulate GM and improve metabolic function, existing research faces several challenges. Firstly, the effects of different strains on host metabolism and the immune system vary widely, but most clinical trials use a limited variety of strains and lack disease-specific screening criteria. For example, certain Lactobacillus and Bifidobacterium species improve hepatic steatosis and bone density in animal models but are less effective in human clinical trials, possibly related to strain specificity. Secondly, there is a lack of standardisation of probiotic doses and treatment duration. There is a wide range of dosages in clinical trials (typically 10^^^9 to 10^^^11 CFU/day), variable treatment durations, and a lack of optimised regimens. In terms of dietary interventions, although high-fibre diets, low-fat diets and Mediterranean diets are beneficial for metabolic health and bone mineral density, specific mechanisms and implementation strategies need to be optimised. Also, the issue of long-term compliance is one of the challenges. Future research could focus on screening specific probiotic strains through macrogenomics and metabolomics; conducting large-scale clinical trials to determine optimal dosages and regimens; designing personalised dietary interventions and incorporating individual metabolic profiles; and strengthening multidisciplinary collaborations for comprehensive clinical trials. After addressing these challenges, probiotics and dietary interventions are expected to be effective strategies for the prevention and treatment of MASLD and OP. Existing NAFLD clinical studies provide an important reference for MASLD, but as the latter is more precisely defined, patients are usually accompanied by more severe metabolic disturbances and systemic inflammation, and studies related to patients with NAFLD may include some patients without significant metabolic dysfunction, this makes the direct applicability of the results of the existing NAFLD studies need to be carefully assessed.

## 4 Conclusion

GM serves as a critical link between MASLD and OP through mechanisms such as systemic inflammation, insulin resistance, and impaired intestinal barrier function. These shared pathways highlight the interconnected nature of two diseases, with GM dysregulation exacerbating both conditions. While interventions targeting GM, including probiotics and dietary modifications, show promise, challenges such as strain specificity and treatment optimization remain. Future research should focus on elucidating these mechanisms further and developing targeted therapies to harness the potential of GM modulation in managing MASLD and OP.
